# Intestinal Injury Biomarkers Predict Mortality in Pediatric Severe Malaria

**DOI:** 10.1128/mbio.01325-22

**Published:** 2022-09-07

**Authors:** Maithri L. Sarangam, Ruth Namazzi, Dibyadyuti Datta, Caitlin Bond, Charles P. B. Vanderpool, Robert O. Opoka, Chandy C. John, Andrea L. Conroy

**Affiliations:** a University of Washington, Seattle, Washington, USA; b Department of Paediatrics and Child Health, Makerere University College of Health Sciences, Kampala, Uganda; c Global Health Uganda, Kampala, Uganda; d Department of Pediatrics, Indiana University School of Medicine, Indianapolis, Indiana, USA; e Center for Global Health, Indiana University School of Medicine, Indianapolis, Indiana, USA; University of Washington

**Keywords:** intestinal injury, severe malaria, acute kidney injury, acidosis, TFF3, I-FABP, mortality, pediatrics, global health

## Abstract

Severe malaria (SM) increases the risk of invasive bacterial infection, and there is evidence to suggest increased gastrointestinal permeability. Studies have shown sequestration of infected erythrocytes in intestinal microvasculature, and *in vivo* studies of rectal mucosa have demonstrated disruption of microvascular blood flow. However, the extent of intestinal injury in pediatric malaria is not well characterized. In this study, two serum biomarkers of intestinal injury, trefoil factor 3 (TFF3) and intestinal fatty acid binding protein (I-FABP), were analyzed in 598 children with SM and 120 healthy community children (CC), 6 months to 4 years of age. Serum was collected at enrollment and 1 month for laboratory studies, and participants were monitored for 12 months. Intestinal injury biomarkers were significantly elevated in children with SM, with 18.1% having levels of TFF3 and/or I-FABP greater than the 99th percentile of CC levels. TFF3 levels continued to be elevated at 1 month, while I-FABP levels were comparable to CC levels. Both markers predicted in-hospital mortality {odds ratio (OR) (95% confidence interval [CI]), 4.4 (2.7, 7.3) and 2.3 (1.7, 3.1)} for a natural log increase in TFF3 and I-FABP, respectively. TFF3 was also associated with postdischarge mortality (OR, 2.43 [95% CI, 1.1, 4.8]). Intestinal injury was associated with acute kidney injury (AKI), acidosis (*P* < 0.001 for both), and angiopoietin 2, a maker of endothelial activation. In conclusion, intestinal injury is common in pediatric severe malaria and is associated with an increased mortality. It is strongly associated with AKI, acidosis, and endothelial activation.

## INTRODUCTION

Malaria remains a significant cause of childhood morbidity and mortality (1). Children with Plasmodium falciparum malaria are at an increased risk of invasive bacterial infections from enteric Gram-negative organisms, particularly nontyphoidal Salmonella (2). Disruptions to the intestinal barrier or increases in barrier permeability can facilitate the translocation of enteric pathogens—or their products—into the systemic circulation, where they can result in immune activation or endothelial dysfunction and can exacerbate multiorgan dysfunction (3, 4).

Multiple studies have reported microvascular sequestration of parasitized erythrocytes in the gut of patients with severe malaria (SM), leading to altered mucosal blood flow, and intestinal injury (5–7). In adults with SM, elevated levels of microbially derived acids in the blood correspond to reduced gut barrier integrity, and reduced clearance of microbial acids is associated with acute kidney injury (AKI) and fatal outcomes in SM (8). AKI is common in pediatric SM, occurring in 24 to 59% of children, and is associated with increased mortality during hospitalization and in the postdischarge period (9).

Intestinal barrier dysfunction occurs through direct injury to the intestinal epithelial cells or disruption of intercellular junctions (10). In the context of SM, intestinal barrier dysfunction may occur as a result of reduced intestinal perfusion as a result of the sequestration of parasitized erythrocytes in intestinal capillaries (5, 6), reduced perfusion secondary to hypotension and systemic inflammation as seen in sepsis (11), or endothelial or immune activation (12). Markers of intestinal injury and dysfunction include intestinal fatty acid binding protein (I-FABP), a marker of enterocyte damage, and trefoil factor 3 (TFF3), a secreted protein produced by goblet cells in the small intestine and colon to maintain mucosal integrity and promote mucosal repair (13). Other markers of immune activation and bacterial translocation include lipopolysaccharide (LPS) binding protein (LBP), which has been used as a marker of endotoxemia (14, 15). The CD14 receptor on monocytes binds to the LPS-LBP complex to facilitate its clearance from blood. In the context of LPS-induced activation of monocytes, soluble CD14 (sCD14) is released through proteolytic cleavage of the membranous form or from intracellular pools and correlates with bacterial products in critical illness, including LPS (15).

We hypothesized that children with severe malaria experience intestinal dysfunction associated with endothelial activation and that intestinal dysfunction is associated with AKI, acidosis, and increased mortality during hospitalization and over follow-up. We evaluated this hypothesis in a prospective cohort study of children hospitalized with severe malaria and community children (CC) over 1 year of follow-up.

## RESULTS

### Description of the study population.

**FIG 1:**
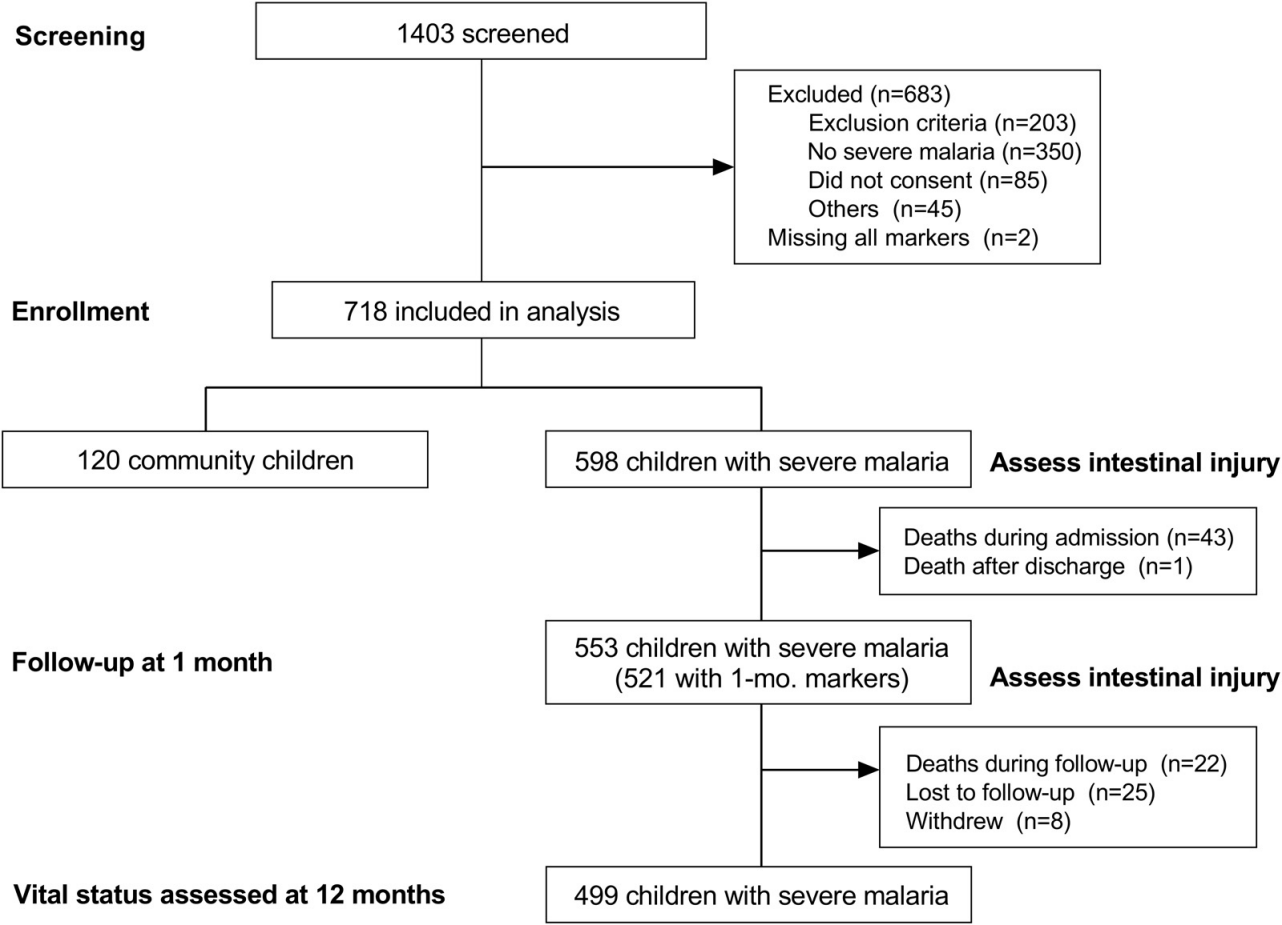
Study flow diagram. Of 1,403 children screened for eligibility, a total of 720 children were enrolled in the study and samples were available for 718 children on admission. At 1 month of follow-up, 521 children with severe malaria returned for an interim health assessment and had samples available to assess markers of intestinal injury. At 12 months, 499 children completed follow-up.

Between 2014 and 2017, 1,403 children were screened for eligibility and 720 children were enrolled in the study (Fig. 1). Data were available for 598 children with SM and 120 community children (CC). The children with SM and CC were comparable in age, sex, and socioeconomic status, and enrollment was equally distributed by sites (Table 1). Children with SM had height-for-age z scores comparable to those of the CC but lower weight-for-age and weight-for-height z scores on enrollment (Table 1). Among the CC, asymptomatic malaria occurred in 11.0% of children as determined by blood smear, 25.2% of children by rapid diagnostic test (RDT), and 27.5% of children by PCR.

**TABLE 1:** Characteristics of study participants^*a*^

Parameter	Value for:		*P* value
	Severe malaria (*n* = 598)	Community children (*n* = 120)	
Demographic characteristics			
Age, in yrs	2.1 (0.92)	2.2 (1.0)	0.45
Sex, no. (%) female	261 (43.7)	56 (46.7)	0.54
Site, no. (%) Kampala	331 (55.4)	67 (55.8)	0.92
Wt-for-age z score	−1.1 (1.1), 591	−0.47 (1.1)	<0.001
Ht-for-age z score	−1.1 (1.3)	−1.26 (1.3)	0.24
Wt-for-ht z score	−0.68 (1.1), 591	0.30 (1.0)	<0.001
Laboratory findings			
WBCs, ×10^3^/μL	12.1 (8.7, 19.2), 595	9.9 (7.8, 11.6), 118	<0.001
Hemoglobin, g/dL	5.7 (3.6, 8.6), 595	11.1 (10.1, 11.8), 118	<0.001
Platelet count, ×10^3^/μL	113 (62, 216), 595	394 (281, 486), 118	<0.001
Glucose, mmol/L	6.9 (5.5, 8.5), 594		
Lactate, mmol/L	4.2 (2.4, 7.6), 573		

a Data presented are means (SD) unless otherwise indicated for demographic characteristics or medians (IQR) for laboratory findings. Statistical analysis done using Student’s *t* test for continuous demographic characteristic measures, Wilcoxon rank sum test for continuous laboratory findings measures, or Pearson’s chi-square test for frequency data. For measures with incomplete data, the number of observations is noted.

Among children with SM and markers of intestinal injury assessed, 43/598 (7.2%) of children with SM died during hospitalization, with 31/43 (72%) dying within the first 24 h. Among SM survivors who remained in the study, 23/522 (4.4%) of children died during follow-up, with 3/23 (13.0%) dying within the first 3 months, 8/23 (34.8%) dying between 3 and 6 months, and the remainder of children dying after 6 months.

### Measures of intestinal injury and dysfunction in children with severe malaria.

**FIG 2:**
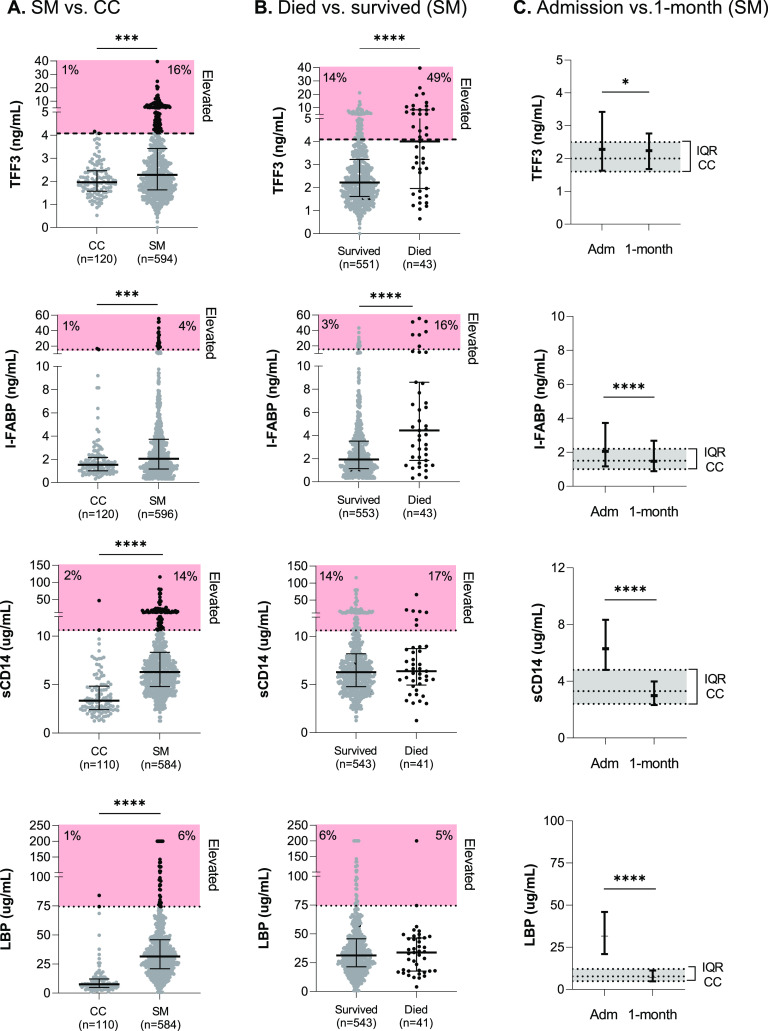
Biomarkers of intestinal injury in community children compared to children with severe malaria at admission, based on mortality, and at 1 month of follow-up. (A) Scatterplots depicting plasma (TFF3, sCD14, and LBP) or serum (I-FABP) levels of biomarkers on admission in community children (CC) or children with severe malaria (SM). Black lines and error bars represent medians and interquartile ranges. The horizontal dotted line depicts biomarker levels that are >99th percentile for the CC, and data points outside the expected population range are depicted in black, with the percentage of children who fall into this category shown in red boxes. Differences between groups were compared using the Wilcoxon rank sum test. (B) Scatterplots depicting biomarkers on admission in children with severe malaria based on survival status. Differences between groups were compared using the Wilcoxon rank sum test. (C) Line graphs depicting the median (interquartile range [IQR]) of biomarker levels in children with severe malaria on admission (Adm) or at 1 month of follow-up (1-month; *n* = 521). The gray shading and dotted lines represent the median and interquartile range for the community children on enrollment. Differences between time points were compared using the Wilcoxon matched-pairs signed rank test. *, *P* < 0.05; ***, *P* < 0.001; ****, *P* < 0.0001.

To evaluate whether there were differences in markers of intestinal injury and dysfunction in children with SM, we compared levels of biomarkers on admission in children with SM compared to CC (Fig. 2). Levels of TFF3, I-FABP, sCD14, and LBP were higher in children with SM than in CC (*P* < 0.001 for all). We further evaluated the biomarkers in the CC based on the presence of malaria parasites to determine whether the elevations observed reflected disease severity or malaria infection. There were no differences in the median levels of TFF3 or I-FABP in the CC irrespective of the method used to diagnose malaria (i.e., blood smear, RDT, or PCR) (*P* > 0.05 for all [Table S1]).

10.1128/mbio.01325-22.10 TABLE S1 Markers of intestinal injury in community children with asymptomatic malaria. Download Table S1, DOCX file, 0.01 MB. Copyright © 2022 Sarangam et al. 2022 Sarangam et al. https://creativecommons.org/licenses/by/4.0/ This content is distributed under the terms of the Creative Commons Attribution 4.0 International license.

Using the 99th percentile of the CC to define a population-specific normal range, the percentages of children with SM with biomarker levels above this threshold were 16% (TFF3), 4% (I-FABP), 14% (sCD14), and 6% (LBP) (Fig. 2A). Overall, 108/598 (18.1%) of children with SM met our definition of intestinal injury, defined as TFF3 and/or I-FABP levels greater than the 99th percentile of CC values, for the respective marker. LBP and sCD14 levels were comparable in children who met our definition of intestinal injury (*P* > 0.05). In children with SM, TFF3 and I-FABP levels were higher in those who died during hospitalization than in those who survived, while LBP and sCD14 levels were comparable (Fig. 2).

To evaluate how intestinal injury changes with clinical recovery, we compared biomarker levels at admission and the 1-month follow-up in children with SM. The levels of I-FABP (marker of enterocyte injury), sCD14, and LBP were reduced relative to the admission levels (*P* < 0.0001, Wilcoxon signed-rank matched-pairs test) and were comparable to those in the CC. However, at the 1-month follow-up, TFF3 (marker of mucosal repair) remained elevated above community levels (Fig. 2).

### Clinical and laboratory complications associated with intestinal injury.

Children with intestinal injury were comparable in age, sex, and height for age to children without intestinal injury (*P* > 0.05 [Table 2]). Weight-for-age and weight-for-height z-scores were significantly lower in children with intestinal injury (*P* < 0.05 [Table 2]). There were no differences in comorbid infections diagnosed in children based on the presence of intestinal injury, with comparable rates of blood culture positivity, stool helminth infection, and HIV positivity, nor were there differences in plasma histidine-rich protein 2 (HRP-2) levels as a measure of parasite biomass (*P* > 0.05 [Table 2]). Children with intestinal injury were more likely to report a history of diarrhea and had clinical signs consistent with increased disease severity, including dehydration, tachycardia, reduced consciousness, jaundice, and AKI. Further, children with intestinal injury had lower hemoglobin and higher leukocyte (WBC) counts and lactate than did children without intestinal injury (*P* < 0.001 [Table 2]).

**TABLE 2:** Clinical and laboratory characteristics on admission in children with severe malaria, according to intestinal injury^*a*^

Parameter	Value for:				*P* value
	No. (% missing)	Combined (*n* = 598)	Intestinal injury (*n* = 108)	No intestinal injury (*n* = 490)	
Demographic characteristics					
Age, in yrs	598 (0)	2.1 (0.92)	2.1 (0.86)	2.1 (0.93)	0.36
Sex, no. (%) female	598 (0)	261 (43.7)	52 (48.2)	209 (42.7)	0.30
Wt-for-age z score	591 (1.2)	−1.1 (1.1)	−1.4 (1.1)	−1.0 (1.1)	0.001
Ht-for-age z score	598 (0)	−1.1 (1.3)	−1.3 (1.2)	−1.1 (1.3)	0.03
Wt-for-ht z score	591 (1.2)	−0.68 (1.1)	−0.94 (1.3)	−0.62 (1.1)	0.01
Admission characteristics					
Symptoms					
History of fever, days	587 (1.8)	3.6 (2.3)	3.7 (2.3)	3.6 (2.3)	0.80
Diarrhea, no. (%)	598 (0)	115 (19.2)	31 (38.7)	84 (17.1)	0.01
Vomiting, no. (%)	598 (0)	316 (52.8)	67 (62.0)	249 (50.8)	0.03
Convulsions, no. (%)	598 (0)	301 (50.3)	43 (39.8)	258 (52.7)	0.02
Clinical signs					
Temp, °C	598 (0)	37.7 (1.2)	37.3 (1.3)	37.8 (1.2)	<0.001
Pulse, beats/min	598 (0)	158 (23)	153 (25)	159 (23)	0.02
Tachycardia, no. (%)	598 (0)	442 (73.9)	72 (66.7)	370 (75.5)	0.06
Respiratory rate, breaths/min	598 (0)	51 (13)	54 (14)	50 (13)	0.002
Tachypnea, no. (%)	596 (0.33)	596 (99.7)	108 (100)	488 (99.6)	1.00
Systolic blood pressure, mm Hg	589 (1.5)	98 (13)	96 (13)	98 (13)	0.14
Blantyre coma score, no. (%)					
0	597 (0.17)	9 (1.5)	4 (3.7)	5 (1.0)	0.005
1		27 (4.5)	9 (8.3)	18 (3.7)	
2		57 (9.6)	14 (13.0)	43 (8.8)	
3		62 (10.4)	14 (13.0)	48 (9.8)	
4		50 (8.4)	12 (11.1)	38 (7.8)	
5		392 (65.7)	55 (51.0)	337 (68.9)	
Shock, no. (%)	588 (1.7)	49 (8.3)	17 (16.2)	32 (6.6)	0.001
Temp gradient, no. (%)	596 (0.33)	34 (5.7)	12 (11.2)	22 (4.5)	0.01
Dehydration, no. (%)	598 (0)	70 (11.7)	25 (23.2)	45 (9.2)	<0.001
Jaundice, no. (%)	597 (0.17)	137 (23.0)	41 (38.3)	96 (19.6)	<0.001
Laboratory findings (median, IQR)					
Hemoglobin, g/dL	595 (0.50)	5.7 (3.6–8.6)	4.2 (2.8–6.7)	6.1 (4.0–8.9)	<0.001
Glucose, mmol/L	594 (0.67)	6.9 (5.5–8.5)	7.1 (4.5–8.9)	6.9 (5.6–8.5)	0.46
Lactate, mmol/L	573 (4.2)	4.2 (2.4–7.6)	5.9 (2.9–13.2)	4.0 (2.3–6.7)	<0.001
WBCs, ×10^3^/μL	595 (0.50)	12.1 (8.7–19.2)	20.0 (12.2–32.7)	11.1 (8.3–17.3)	<0.001
Platelets, ×10^3^/μL	595 (0.50)	113 (62–216)	147 (81–270)	104 (58–212)	0.01
Peripheral parasite density, parasites/μL	598 (0)	13,434 (0–135,627)	2,928 (0–54,121)	19,645 (49–158,019)	0.001
Plasma HRP-2, ng/mL	593 (0.84)	2,372 (364–5,898)	1,929 (204–4,763)	2,407 (433–6,220)	0.42
Comorbid diagnoses					
Blood culture positive, no. (%)	584 (2.3)	22 (3.8)	4 (3.7)	18 (3.8)	1.00
Gram negative, no. (%)	22	15 (68.2)	2 (50)	13 (72.2)	0.57
Gram positive, no. (%)	22	7 (31.8)	2 (50)	5 (27.8)	0.57
Stool helminth, no. (%)	480 (19.7)	17 (3.5)	3 (4.1)	14 (3.4)	0.73
Gastroenteritis, no. (%)	598 (0)	88 (14.8)	26 (24.1)	62 (12.7)	0.004
HIV, no. (%)	594 (0.67)	13 (2.2)	2 (1.9)	11 (2.3)	1.00
Medication history					
Prior antibiotics, no. (%)	588 (1.7)	203 (34.5)	47 (43.9)	156 (32.4)	0.02
Prior antimalarial, no. (%)	588 (1.7)	364 (61.9)	66 (61.7)	298 (62.0)	0.96

a Data are presented are means (SD) unless otherwise indicated. Intestinal injury versus no intestinal injury compared using Student’s *t* test for Gaussian continuous measures or Wilcoxon rank sum test for non-Gaussian continuous measures (i.e., laboratory findings), and chi-square test or Fisher’s exact test, as appropriate, for categorical measures.

**FIG 3:**
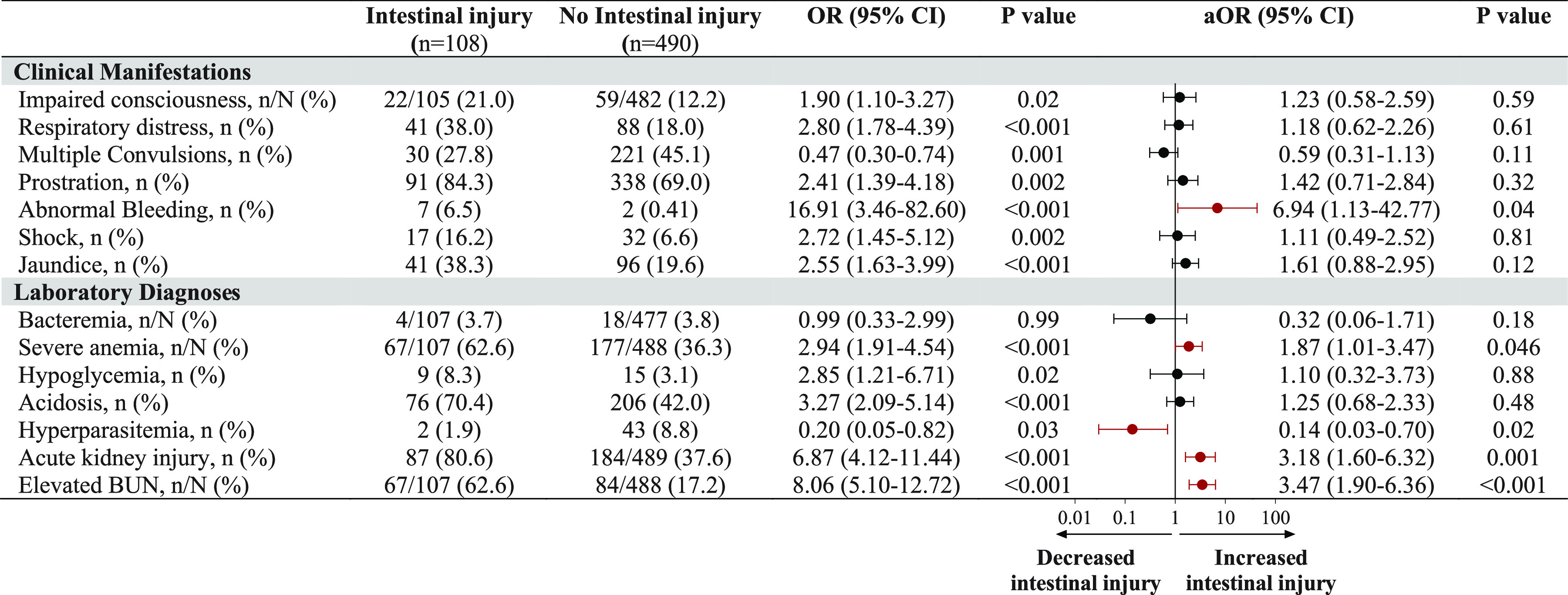
Associations between clinical complications with intestinal injury. Shown are forest plots depicting the adjusted odds ratio (OR) and 95% confidence interval (CI) from logistic regression models with intestinal injury at enrollment and clinical manifestations of severe malaria as well as laboratory diagnosis. Unadjusted OR and *P* values are shown to the left of the forest plot, and the adjusted OR (aOR), adjusted for all complications, age, sex, and study site, is shown to the right. Significant results are presented in red.

**FIG 4:**
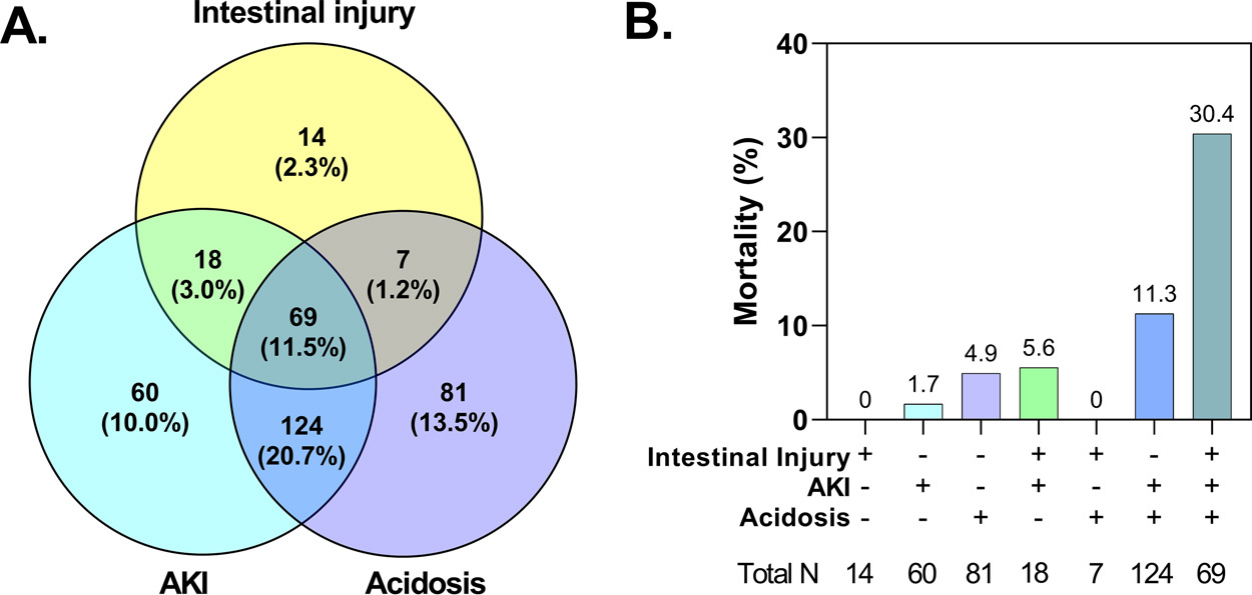
Interrelationships between acute kidney injury (AKI), acidosis, and intestinal injury and mortality. (A) Venn diagram showing the number and frequency of children with each complication among the 596 children with acute kidney injury, acidosis, and intestinal injury defined. (B) Bar graph showing the frequency of in-hospital mortality corresponding to each section of the Venn diagram based on the presence of AKI, acidosis, and intestinal injury.

To evaluate which clinical and laboratory features were associated with the presence of intestinal injury, we conducted multivariable logistic regression analyses including key clinical manifestations and laboratory diagnoses, adjusting for participant age, sex, and site. Abnormal bleeding, severe anemia, AKI, and elevated blood urea nitrogen (BUN) were independent predictors of intestinal injury (Fig. 3). Based on these findings and previous studies reporting relationships between AKI, acidosis, and translocation of bacterial products in adults with SM, we evaluated the interrelationships between these complications in children with SM (Fig. 4). Overall, 11.5% of children (*n* = 69/598) presented with intestinal injury, AKI, and acidosis.

### Relationship between intestinal injury and survival among children with SM.

To evaluate whether intestinal injury modified the risk of mortality in children with SM, we examined mortality in children based on the presence of intestinal injury, AKI, and acidosis. Children with only one of the three complications alone (e.g., intestinal injury without AKI or acidosis) had relatively low mortality, but the presence of AKI and acidosis in the context of intestinal injury was associated with 30% mortality (Fig. 4A). In children with elevations in both TFF3 and I-FABP levels, mortality was 60% (*n* = 6/10).

**FIG 5:**
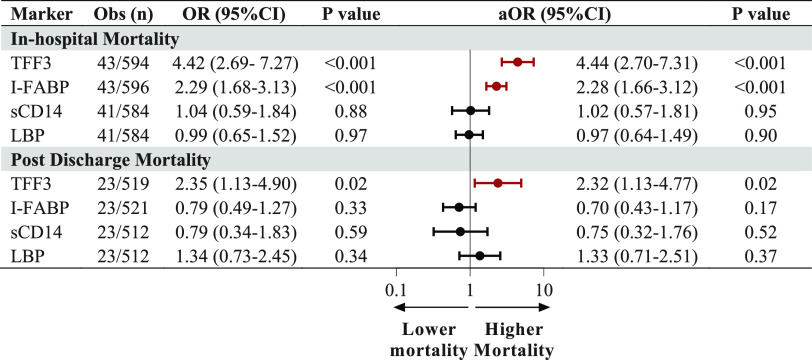
Associations between biomarkers of intestinal injury and mortality during hospitalization and following hospital discharge. Forest plots depicting the aOR and 95% CI, using logistic regression models with natural log-transformed biomarkers levels on admission, for in-hospital and postdischarge mortality. Models were adjusted for age, sex, and study site. Significant results are depicted in red.

To further examine the relationship between intestinal injury and mortality in SM, we evaluated markers of intestinal injury and dysfunction separately. There were significant increases in both TFF3 and I-FABP in children who died during the initial admission compared to survivors (*P* < 0.001 for both [Fig. 5]). There were no differences in LBP and sCD14 in children who died compared to children who survived. A 1-unit increase in natural log-transformed biomarkers was associated with adjusted odds ratios (ORs) of 4.4 (95% confidence interval [CI], 2.7 to 7.3) and 2.3 (95% CI, 1.7 to 3.1) for TFF3 and I-FABP, adjusting for age, sex, and site (Fig. 5).

To determine whether intestinal injury was associated with worse postdischarge complications over 12 months of follow-up, we evaluated biomarkers of intestinal injury and postdischarge mortality. Median levels of TFF3 (*P* = 0.02), but not I-FABP (*P* = 0.18), were higher at admission among children who died during follow-up, corresponding to adjusted odds ratios of 2.3 (95% CI, 1.1 to 4.8) and 0.7 (95% CI, 0.4 to 1.2) for TFF3 and I-FABP, adjusting for participant age, sex, and site (Fig. 5). There were no differences in levels of intestinal biomarkers at 1 month of recovery in children who subsequently died (*P* > 0.05).

### Intestinal injury markers and SM pathophysiology.

**FIG 6:**
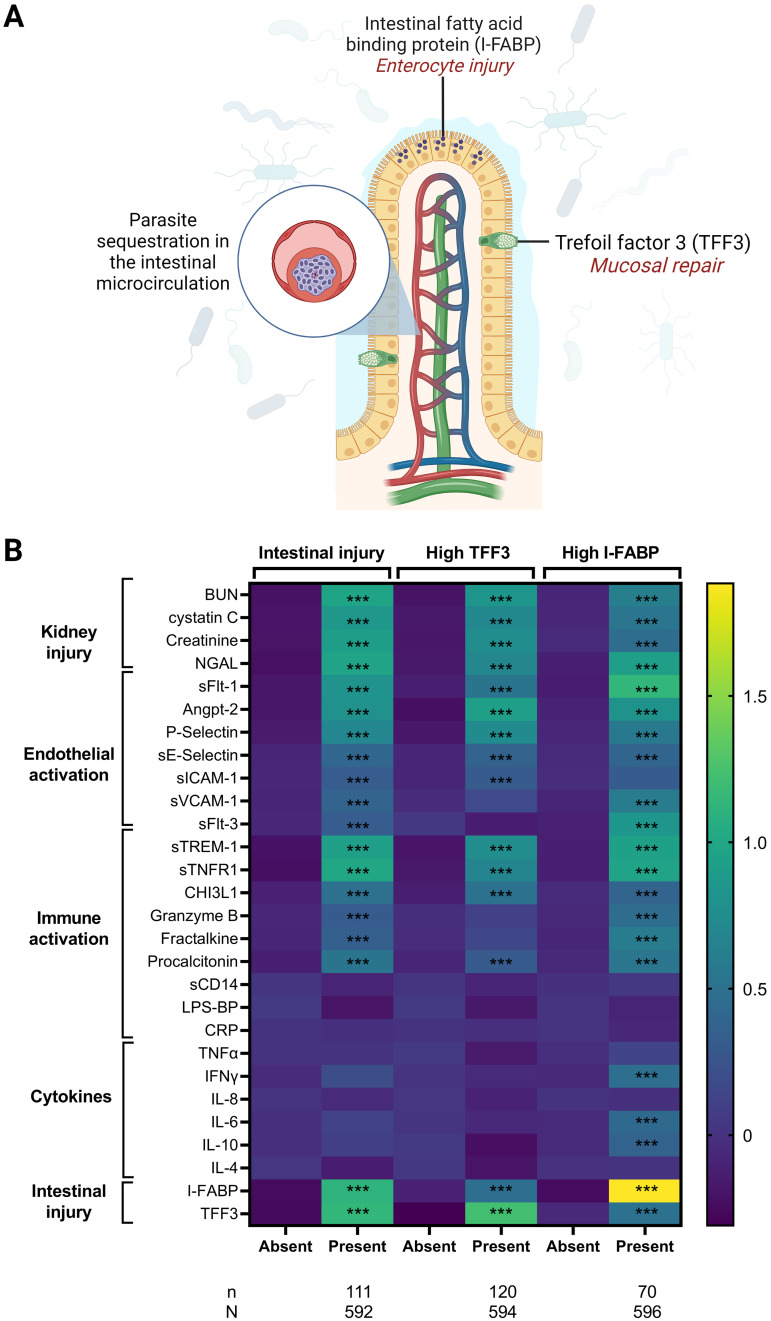
Relationship between biomarkers of intestinal injury and measures of kidney injury, endothelial activation, and inflammation. (A) Diagram of intestinal villus depicting enterocytes expressing I-FABP and goblet cells secreting TFF3. (B) Shown is a heat map depicting the mean standardized biomarker levels based on the presence of intestinal injury, defined as high trefoil factor 3 (TFF3) or intestinal fatty acid binding protein (I-FABP) levels above the reference level. The relationship between pathways of kidney injury (blood urea nitrogen [BUN], cystatin C, creatinine, neutrophil gelatinase-associated lipocalin [NGAL]), endothelial activation (soluble fms-related receptor tyrosine kinase 1 [sFlt-1], angiopoietin 2 [Angpt-2], P-selectin, soluble E-selectin [sE-Selectin], soluble ICAM-1 [sICAM-1], soluble VCAM-1 [sVCAM-1], soluble fms-related receptor tyrosine kinase 3 [sFlt-3]), immune activation (soluble triggering receptor expressed on myeloid cell-1 [sTREM-1], soluble TNF receptor 1[sTNFR1], chitinase-3-like protein 1 [CHI3L-1], granzyme B, fractalkine, procalcitonin, soluble CD-14 [sCD14], lipopolysaccharide binding protein [LPS-BP], c-reactive protein [CRP]), cytokines (tumor necrosis factor alpha [TNF-α], interferon gamma [IFN-γ], interleukin 8 [IL-8], IL-6, IL-10, IL-4) and intestinal injury (I-FABP, TFF3) are presented based on the presence or absence of intestinal injury, high TFF3, and high I-FABP. Relationships significant following adjustment for multiple comparisons using Bonferroni correction are indicated by triple asterisks.

There are multiple potential mechanisms through which SM may lead to intestinal injury. One possibility involves sequestration of parasitized erythrocytes in the mucosal microvasculature leading to local hypoxia and tissue injury. There was no difference in total parasite biomass with intestinal injury (Table 2). We further evaluated the relationship between intestinal injury and markers of kidney injury, endothelial activation, and inflammation (Fig. 6).

Overall, TFF3 and I-FABP were correlated with markers of kidney injury, including markers of functional injury and decreased glomerular filtration (creatinine, BUN, and cystatin C), structural injury (neutrophil gelatinase-associated lipocalin [NGAL]), and inflammation (soluble tumor necrosis factor [TNF] receptor 1 [sTNFR1]), with the relationships between TFF3 (mucosal repair) stronger than those observed for I-FABP (enterocyte injury). Similarly, both TFF3 and I-FABP were positively correlated with markers of endothelial activation and dysfunction. While angiopoietin 2 (Angpt-2) and P-selectin were associated with TFF3, I-FABP was correlated with soluble Fms-related receptor tyrosine kinase 1 (sFlt-1) as well as soluble vascular cell adhesion molecule 1 (sVCAM-1) in children with acidosis. There was no relationship between the marker of mucosal repair, TFF3, and measures of systemic inflammation, while the marker of enterocyte injury, I-FABP, was positively correlated with inflammatory markers procalcitonin, TNF-α, interferon gamma (IFN-γ), interleukin-6 (IL-6), and interleukin-10 (IL-10).

## DISCUSSION

**FIG 7:**
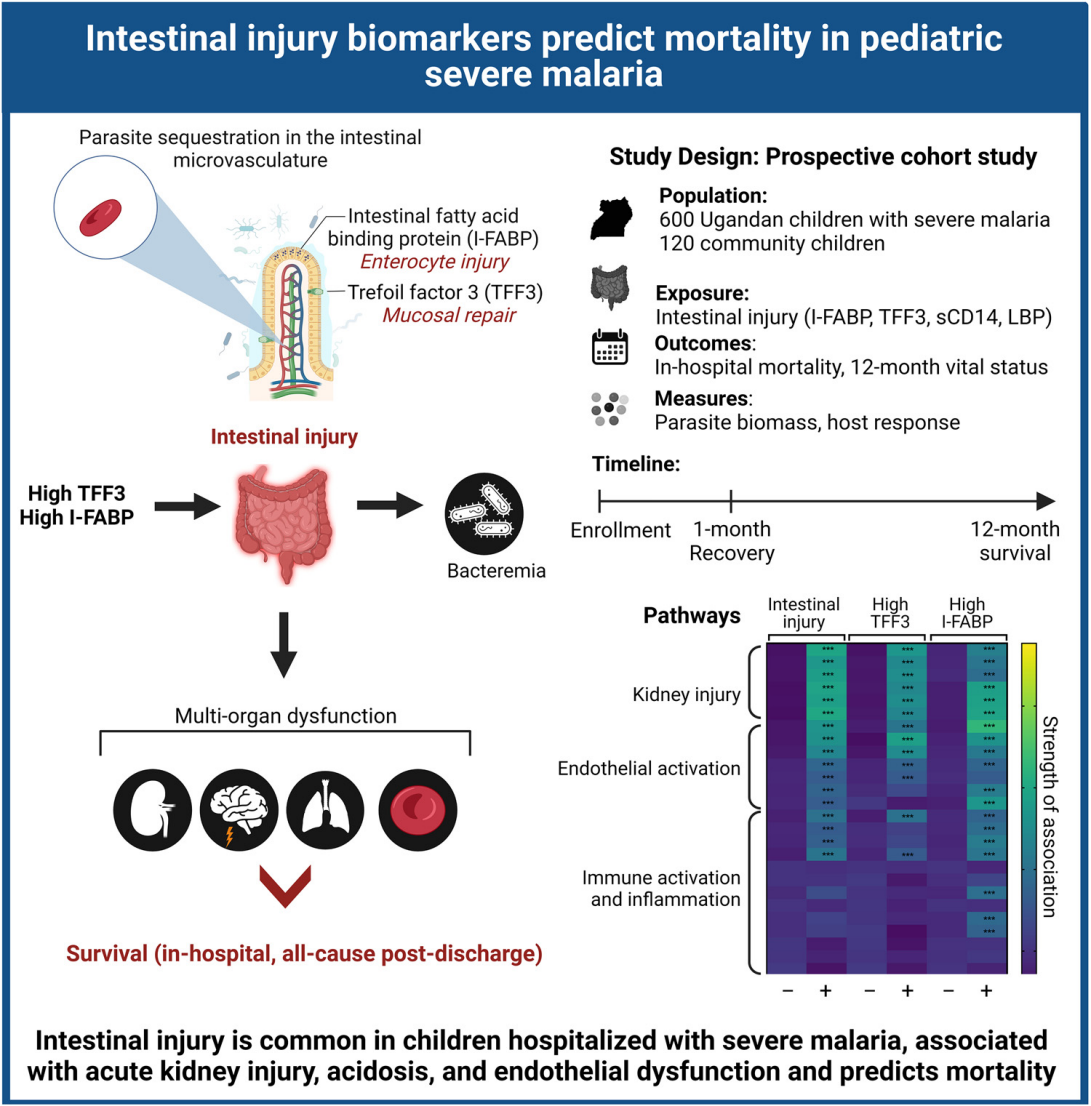
Conceptual diagram of intestinal injury in severe malaria.

There is a growing body of evidence supporting a role for intestinal injury in severe malaria pathogenesis (7, 16, 17). In the present study, we assessed intestinal injury in a well-characterized cohort of children with severe malaria and report elevated levels of markers of intestinal injury (TFF3 and I-FABP) that interacted with AKI and acidosis to increase in-hospital mortality. Among survivors at 1 month of follow-up, there was evidence of ongoing mucosal healing with increased TFF3 levels relative to those in community children. Increased TFF3 was associated with increased risk of postdischarge mortality over 1 year of follow-up. The results are conceptually summarized in Fig. 7.

TFF3 is a stable protease-resistant protein secreted by mucin-producing cells onto the mucosal surface, where it protects the intestinal epithelium from injury. In response to injury, it is upregulated at the site of injury to promote cell migration, reduce intestinal permeability, inhibit apoptosis, and thus support epithelial restitution (18). Deletion of the TFF3 gene results in exquisite sensitivity to intestinal injury, resulting in mortality due to an inability to repair the epithelium (19). TFF3 is upregulated in the context of inflammatory bowel disease, colitis, and sepsis (20, 21). In patients with sepsis, TFF3 levels were associated with kidney injury, multiorgan dysfunction, and mortality (21). In patients following gastrointestinal surgery, TFF3 levels rose steadily, supporting its role in intestinal recovery (21). In the present study, 16% of children with SM had TFF3 levels on admission above the upper limit of normal for the population and TFF3 levels remained higher than those of community children at 1 month of follow-up, supportive of ongoing mucosal healing.

I-FABP, a marker of enterocyte damage, is rapidly released into the circulation following injury to the small intestine. It is produced by enterocytes, with higher concentrations closer to the tips of intestinal villi (22). Elevated I-FABP was less frequent in the study population than TFF3, with only 4% of children having I-FABP levels above the 99th percentile established using community children. Among children with SM, I-FABP levels returned to levels seen in community children by 1 month of follow-up, suggesting no ongoing intestinal injury at that time. These patterns, and the stronger association between I-FABP and markers of inflammation, suggest that I-FABP may be elevated only shortly after the injury occurs, while TFF3 continues to be elevated through the course of healing. Similar to TFF3, I-FABP was elevated in children with AKI and acidosis as well as children with hypoglycemia and hypoxemia on admission. Consistent with its role as a marker of intestinal damage, I-FABP was positively correlated with markers of inflammation (procalcitonin, TNF-α, and IL-6) and markers of systemic endothelial activation (sVCAM-1, sE-selectin, Angpt-2, P-selectin, and sFlt-1).

Our study demonstrates that there is significant overlap in children with biomarker evidence of intestinal injury, AKI, and acidosis with a corresponding increase in mortality in children with all three features present. Prior studies have identified microbial acids as important contributors to acidosis in severe malaria associated with increased risk of mortality (8, 23). These findings suggest that either bacteria or their products are able to cross the intestinal epithelial barrier. Although AKI can lead to altered metabolism of acids and to worsening acidosis, there is also evidence that acids elevated in severe malaria may be nephrotoxic and contribute to AKI (24).

Intestinal barrier dysfunction may allow for translocation of bacterial products into the blood, including LPS, which is a potent proinflammatory mediator (25). LPS is challenging to measure in serum, so LBP and sCD14 were used as surrogate markers to indicate the presence of endotoxin or other bacterial products (14, 15). LPS binds LBP, which then forms a complex with either membrane bound CD14 or sCD14. While both LBP and sCD14 correlate with LPS, they lack specificity (26, 27). The lack of association with intestinal injury suggests that the effects of intestinal injury in this cohort may not be due to microbial translocation or translocation of bacterial products that cause elevations in LBP and sCD14. This finding is supported by a study looking at endotoxemia in pediatric severe malaria patients, using an endotoxin activity assay in fresh whole blood (17). That study found no correlation between endotoxemia and I-FABP, nor did it find an association between endotoxemia and mortality (17). While the study findings do not support increased translocation of bacteria into the blood based on LPS and sCD14, this does not exclude the possibility of translocation of bacterial products, including acids, via disruption of intestinal tight junctions.

The association between intestinal injury and acidosis may occur through a common pathway of tissue hypoxia contributing to hypoperfusion and injury to the gut. Acidosis and intestinal injury may also be mediated through AKI, as AKI can worsen acidosis and AKI is associated with the accumulation of other products, including blood urea nitrogen (BUN), leading to elevated BUN. Elevated BUN is a frequent complication in severe malaria and occurred in 25% of the study population. Blood urea nitrogen correlated with levels of both TFF3 and I-FABP. Urea is partially excreted through the gut. In the setting of elevated BUN, more urea may be transported into the gut lumen, where it can cause injury to the enterocytes lining the gut wall (22–24, 28). The toxicity of urea is amplified in settings where there is microbial conversion of urea to ammonia (28). Additional studies are needed to evaluate whether there is increased urea in the gut of children with SM, to evaluate how the gut microbiome facilitates the conversion to ammonia and to determine whether differences in the microbiome may relate to increased susceptibility to intestinal injury through the conversion of urea to ammonia.

Increased systemic immune and endothelial activation in severe malaria and the translocation of enteric microbial products in intestinal injury may promote AKI (29). In addition, increased inflammatory cytokines in the setting of AKI may in turn cause intestinal injury through disruption of tight junctions (29). Proinflammatory cytokines, such as TNF-α and IFN-γ, are important regulators of tight junction integrity and can cause decreased levels of tight junction proteins, increasing intestinal permeability (10, 29). Systemic inflammation is also associated with endothelial activation (30). Angpt-2 expression is increased in SM and can potentiate cytokine-triggered effects on the vasculature (31). We have shown that endothelial activation is strongly associated with intestinal injury (30), and systemic endothelial activation is well described for SM and associated with AKI and mortality (32). In our study, the markers of intestinal injury, TFF3 and I-FABP, were significantly correlated with Angpt-2, which promotes a proinflammatory state and microvascular permeability (33). In addition, both markers were significantly correlated with P-selectin and soluble E-selectin, which promote leukocyte motility (33). This endothelial activation may also be caused by localized sequestration of parasitized erythrocytes leading to tissue hypoperfusion injury and local inflammation. Although calculated sequestered parasite biomass was not associated with TFF3 or I-FABP in our study, other studies have reported highly variable and dense sequestration of parasitized erythrocytes in the intestinal vasculature that disrupts microcirculation (5) and leads to endothelial activation (32). As localized differences in sequestration may not reflect total sequestered biomass, future studies should investigate sequestration within the rectal mucosa of children with SM and its relationship with intestinal permeability and markers of intestinal inflammation in stool.

Bacteremia due to enteric pathogens is well described for pediatric severe malaria, and severe malaria is a risk factor for invasive nontyphoidal Salmonella infection (2). In the present study, bacteremia was found in 3.8% of study participants, which is consistent with previous studies in East Africa (34) but lower than other estimates in the region (2). The relatively low prevalence of bacteremia in the study may reflect widespread use of antimicrobial medications in community settings and administration of antibiotics prior to referral. Over one-third of study participants reported taking antibiotics for their current illness, which may have interfered with our ability to detect bacteremia among patients. As intestinal injury may lead to increased intestinal permeability and facilitate microbial translocation to the blood (4), and bacteremia is a well-described risk factor for mortality in pediatric SM (2), additional studies are needed to delineate pathways of intestinal injury and mortality in SM.

Limitations include a lack of urine samples to evaluate whether increased levels of TFF3 and I-FABP in the context of AKI reflect increased production and/or altered clearance by the kidney. Although acidosis was assessed using point-of-care blood gas measures on admission, quantities of organic acids and the breakdown of host versus microbial acids in the population are unknown. This study used blood biomarkers to assess intestinal injury. Future studies should assess markers of intestinal injury in stool samples, which may be useful in differentiating children with intestinal injury secondary to severe malaria from children with a primary gastrointestinal illness in the setting of asymptomatic malaria parasitemia. Finally, while all children met a clinical definition of malaria, we cannot rule out the possibility that some children had incidental parasitemia and another infectious cause of admission.

This study has several strengths, including the size of the cohort, recruitment of community children to establish population reference ranges, and detailed clinical assessment of participants and follow-up over 12 months with greater than 90% retention. In addition, laboratory studies, including blood cultures, were obtained for the majority of study participants. The collection of a blood sample at convalescence at 1 month of follow-up provided insight into the kinetics of intestinal injury and repair in severe malaria. Further, we assessed multiple pathways of immune activation and injury in severe malaria to evaluate how intestinal injury may interact with other complications in severe malaria, including AKI and acidosis, to contribute to increased mortality.

In summary, this study found that intestinal injury occurs in 18.1% of children with severe malaria and is associated with AKI, acidosis, and mortality. Intestinal injury may contribute to worse outcomes by exacerbating acidosis and leading to systemic endothelial and immune activation, and kidney injury. These findings require validation to determine whether they could facilitate improved risk stratification of children with severe malaria and identify children who may benefit from targeted therapies. Additional studies are needed to delineate the mechanisms of intestinal injury in severe malaria and evaluate whether strategies to improve gut integrity (e.g., citrulline and quercetin) (35) may lead to improved recovery and long-term survival following severe malaria.

## MATERIALS AND METHODS

### Study population.

Between 2014 and 2017, we prospectively enrolled 600 children with severe malaria and 120 community children between 6 months to 4 years of age at two sites in Uganda (36). The study was designed to assess cognitive outcomes following severe malaria at 12 months of follow-up in children <5 years of age. Children were recruited at two referral hospitals in central and eastern Uganda: Mulago National Referral Hospital in Kampala and Jinja Regional Referral Hospital in Jinja. Jinja is considered a periurban district and has a higher malaria endemicity than Kampala, an urban setting (37). Children with severe malaria were eligible if they had diagnostic evidence of malaria with either a positive rapid diagnostic test for Plasmodium falciparum histidine-rich protein 2 (HRP-2) or direct visualization of parasites by Giemsa microscopy and any of the five most common severe malaria features in Ugandan children: prostration, severe anemia, multiple seizures, respiratory distress, and coma. Exclusion criteria for children with severe malaria included a history of chronic illness requiring medical care (including known HIV infection), history of coma, head trauma, known developmental delay, cerebral palsy, or prior hospitalization for malnutrition. A delayed exclusion criterion was evidence of meningitis in children with decreased consciousness who had a lumbar puncture performed (leukocyte count > 5 cells/μL). Children with severe malaria were managed according to Ugandan National Treatment guidelines for malaria at the time of the study, which included intravenous artesunate at 3 mg/kg of body weight for the treatment of severe malaria followed by an oral course of artemisinin combination therapy. Complications were appropriately managed per the treatment protocol. Hypoglycemia was managed with glucose boluses, frequent blood glucose checks, and nasogastric tube feed as needed. Dehydration was managed with bolus and maintenance fluids or oral hydration depending on the degree of dehydration. Shock was managed with 20-mL/kg boluses of normal saline, with blood transfusion and antibiotics if the patient was not responsive to three boluses. For AKI, fluid deficits and electrolyte imbalances were corrected, and children were referred for hemodialysis if indicated. Acidosis was managed by treating anemia, if present, and administering sodium bicarbonate for serum bicarbonate levels of <13 mmol/L. Hyperkalemia was treated with nebulized salbutamol or calcium gluconate, depending on the degree of elevation. Convulsions were managed with rectal diazepam, with progression to second- and third-line medications if indicated.

Community children were recruited from the nuclear family, extended family, or household area of children with severe malaria. Inclusion criteria included residence in the same or nearby neighborhood as a child with severe malaria. Exclusion criteria for the community children included an illness requiring medical care within the previous 4 weeks, a major medical or neurologic abnormality at screening physical examination, and an active illness or axillary temperature on screening >37.5°C.

### Study procedures.

On enrollment, children had a complete history and physical exam, including a blood draw for clinical and study-related procedures. Whole blood was collected in additive-free tubes or EDTA-anticoagulated BD Vacutainer tubes for isolation of serum or plasma, respectively. A complete blood count was performed on EDTA-anticoagulated blood on admission. Peripheral blood smears were used to quantify parasite density using Giemsa staining with standard protocols. HIV counseling and testing was conducted for all study participants according to the national testing algorithm. For children <18 months or children with discordant test results, HIV status was assessed using DNA PCR using a College of American Pathologists (CAP)-accredited laboratory. To assess for bacteremia, approximately1 to 3mL of whole blood was inoculated into pediatric blood culture bottles (Peds Plus/F), which were incubated in an automatic Bactec 9050 blood culture system for up to 5 days. Positive samples were Gram stained and subcultured on blood agar, chocolate agar, or MacConkey agar plates. In children with decreased consciousness, a lumbar puncture was performed to assess for meningitis following caregiver consent provided that there were no clinical contraindications (i.e., signs of raised intracranial pressure). Point-of-care tests to guide clinical management included i-STAT to assess a basic metabolic panel and blood gases, lactate, and glucose measurements.

Socioeconomic status was evaluated using a validated scoring instrument that assesses material possessions, house structure, and access to food, water, and electricity (38). At 1 month of follow-up, children with severe malaria were asked to return for an interim health assessment, a blood draw to assess recovery of intestinal and kidney injury, and a neurologic examination. At 12 months of follow-up, children returned for a neuropsychological assessment which did not include a blood draw.

### Sample collection, processing, and storage.

Whole-blood samples in additive-free tubes were left to clot for 60 min at room temperature for serum collection, and whole blood collected in EDTA-anticoagulated BD Vacutainer tubes was used for plasma isolation. Tubes were centrifuged at 1,200 × *g* for 20 min by the local coordinating laboratory for serum and plasma collection. Plasma and serum were transferred to a barcode-labeled cryogenic tube for −80°C storage. Sample aliquots were shipped to the United States on dry ice and sent in batches to the Indiana University Health Pathology Laboratory for chemistry testing. Creatinine was measured on admission, at 24 h, and at 1 month of follow-up using a Beckman Coulter AU5822 chemistry analyzer using the modified Jaffe colorimetric method (Beckman Coulter, Brea, CA). The method is traceable to an isotope dilution mass spectrometry (IDMS) reference method using U.S. National Institute of Standards and Technology (NIST) standard reference material (SRM967). The remaining aliquots were stored at −80°C for future biomarker testing in the CHILD (Child Health ImmunoLogy & Development) lab.

### Defining acute kidney injury and acidosis.

Acute kidney injury was defined using the Kidney Disease: Improving Global Outcomes (KDIGO) criteria based on a 1.5-fold increase in creatinine over estimated baseline or a 0.3-mg/dL change in creatinine within 48 h (39) as described previously (40). Staging was as follows: stage 1, 1.5- to 1.9-fold increase in creatinine over baseline; stage 2, 2.0- to 2.9-fold increase over baseline; and stage 3, ≥3.0-fold increase over baseline. Baseline creatinine was estimated using a height-independent approach to back-calculate creatinine assuming a normal glomerular filtration rate (GFR) of 120 mL/min per 1.73 m^2^ as described previously (41). A pediatric nephrologist was consulted for children with AKI for symptomatic management and referral for hemodialysis as indicated. At the time the study was conducted, hemodialysis was available as a fee-based service on adult wards on a limited basis due to availability. Elevated blood urea nitrogen (BUN) was defined as a BUN of >20 mg/dL.

At 1 month of follow-up, acute kidney disease was defined as a 1.5-fold increase in creatinine over estimated baseline or an estimated glomerular filtration rate (eGFR) of <90 mL per min/1.73 m^2^ using the CKiD creatinine-based GFR estimating equation (39, 42, 43). Acidosis was defined using the WHO definition of acidosis as a feature of severe malaria as a base deficit of >8 mmol/L or, if unavailable, a plasma bicarbonate of <15 mmol/L or venous lactate of >5 mmol/L (44).

### Intestinal and endothelial activation biomarker assessment.

Serum or plasma samples were analyzed for biomarkers of intestinal injury in Uganda on all cryopreserved samples. Children were considered to have intestinal injury if they had either elevated TFF3 or I-FABP compared to the population reference levels (>99th percentile for the population was used as the upper limit of normal). Markers were assessed using a custom Luminex MagPix panel including plasma trefoil factor 3 (TFF3), angiopoietin 2 (Angpt-2), soluble Fms-related receptor tyrosine kinase 1 (sFlt-1), P-selectin, sE-selectin, soluble vascular cell adhesion molecule 1 (sVCAM-1), chitinase-3-like protein 1 (CHI3L1), and procalcitonin (R&D Systems, Minneapolis, MN). A modified cytokine Luminex panel was used to measure serum levels of selected cytokines, including tumor necrosis factor alpha (TNF-α), interferon gamma (IFN-γ), interleukin-8 (IL-8), interleukin-6 (IL-6), interleukin-10 (IL-10), and interleukin-4 (IL-4), vascular endothelial growth factor (VEGF), granzyme B, Fractalkine, and sFlt-3 (R&D Systems, Minneapolis, MN). Intestinal fatty acid binding protein (I-FABP), soluble CD14 (sCD14), LPS binding protein (LBP), C-reactive protein (CRP), and soluble intercellular adhesion molecule 1 (sICAM-1) were measured by enzyme-linked immunosorbent assay (ELISA) using DuoSets by R&D Systems.

### Ethics and consent to participate.

Written informed consent was obtained from the parents or legal guardians of all study participants after emergency clinical care was provided to stabilize children. Ethical approval was granted by the institutional review boards at Makerere University School of Medicine, the University of Minnesota, and Indiana University. The Uganda National Council for Science and Technology approved the study.

### Statistical analysis.

Data were analyzed using STATA v16.1 (StataCorp) and GraphPad Prism v9.0. Nonparametric analyses used for assessing differences in intestinal biomarkers included Wilcoxon rank sum analysis for dichotomous outcomes, Kruskal-Wallis analysis for three or more groups, and a nonparametric test of trend. Categorical variables were analyzed using Pearson’s chi-square test or Fisher’s exact test, as appropriate. For parametric analyses, biomarkers were transformed using the natural log. For multivariable models, logistic regression was used for dichotomous variables and linear regression for continuous variables. Variables included in regression analyses were defined *a priori* based on a hypothesized relationship between the biomarkers and measure of interest. Age, sex, and study site were included in all multivariable models. To evaluate patterns in pathways of host response and injury, biomarkers were standardized to have a mean of zero and standard deviation of one and the standardized means were presented in a heat map. To adjust for multiple comparisons using Bonferroni correction, an adjusted *P* value of 0.0018 was considered significant.

### Data availability.

The data that support the findings of this study are available on request from the corresponding author.
