# Supplementary material for: Intestinal Injury Biomarkers Predict Mortality in Pediatric Severe Malaria
Source: mBio. 2022 Sep 7;13(5):e01325-22. doi: 10.1128/mbio.01325-22 (PMC9601216; doi:10.1128/mbio.01325-22)
Supplement: TABLE S1 [file mbio.01325-22-s0001.docx]

**Supplemental Content**

Sarangam ML, Namazzi R, Datta D, Bond C, Vanderpool CPB, Opoka RO, John CC, Conroy AL. *mBio* 2022. Intestinal injury biomarkers predict mortality in pediatric severe malaria.

Content:

Table S1. Markers of intestinal injury in community children with asymptomatic malaria

**Table S1. Markers of intestinal injury in community children with asymptomatic malaria**

| **Method of diagnosis** | **Malaria negative** | **Malaria positive** | **P value** |
| --- | --- | --- | --- |
| Blood smear | n=105 | n=13 |  |
| I-FABP | 1.54 (1.02, 2.13) | 1.48 (0.99, 1.69) | 0.90 |
| TFF-3 | 1.95 (1.58, 2.46) | 2.10 (1.57, 2.38) | 1.00 |
| Rapid diagnostic test | n=86 | n=29 |  |
| I-FABP | 1.53 (0.92, 2.17) | 1.48 (1.03, 1.89) | 0.91 |
| TFF-3 | 1.99 (1.57, 2.46) | 1.94 (1.62, 2.46) | 0.92 |
| PCR | n=87 | n=33 |  |
| I-FABP | 1.60 (1.02, 2.28) | 1.44 (0.99, 1.89) | 0.66 |
| TFF-3 | 1.94 (1.62, 2.46) | 2.10 (1.51, 2.46) | 0.86 |

Data presented as median (IQR) and analyzed using a Wilcoxon rank sum test
